# Electrochemical Determination of Kynurenine Pathway Metabolites—Challenges and Perspectives

**DOI:** 10.3390/s21217152

**Published:** 2021-10-28

**Authors:** Ilona Sadok, Magdalena Staniszewska

**Affiliations:** Laboratory of Separation and Spectroscopic Method Applications, Centre for Interdisciplinary Research, Faculty of Science and Health, The John Paul II Catholic University of Lublin, 20-708 Lublin, Poland; magdalena.staniszewska@kul.pl

**Keywords:** kynurenine pathway, tryptophan metabolites, kynurenine, electrochemical detection, electrochemical sensors, biological samples analysis

## Abstract

In recent years, tryptophan metabolism via the kynurenine pathway has become one of the most active research areas thanks to its involvement in a variety of physiological processes, especially in conditions associated with immune dysfunction, central nervous system disorders, autoimmunity, infection, diabetes, and cancer. The kynurenine pathway generates several metabolites with immunosuppressive functions or neuroprotective, antioxidant, or toxic properties. An increasing body of work on this topic uncovers a need for reliable analytical methods to help identify and quantify tryptophan metabolites at physiological concentrations in biological samples of different origins. Recent methodological advances in the fabrication and application of electrochemical sensors promise a rise in the future generation of novel analytical systems. This work summarizes current knowledge and provides important suggestions with respect to direct electrochemical determinations of kynurenine pathway metabolites (kynurenines) in complex biological matrices. Measurement challenges, limitations, and future opportunities of electroanalytical methods to advance study of the implementation of kynurenines in disease conditions are discussed.

## 1. Introduction

**Figure 1:**
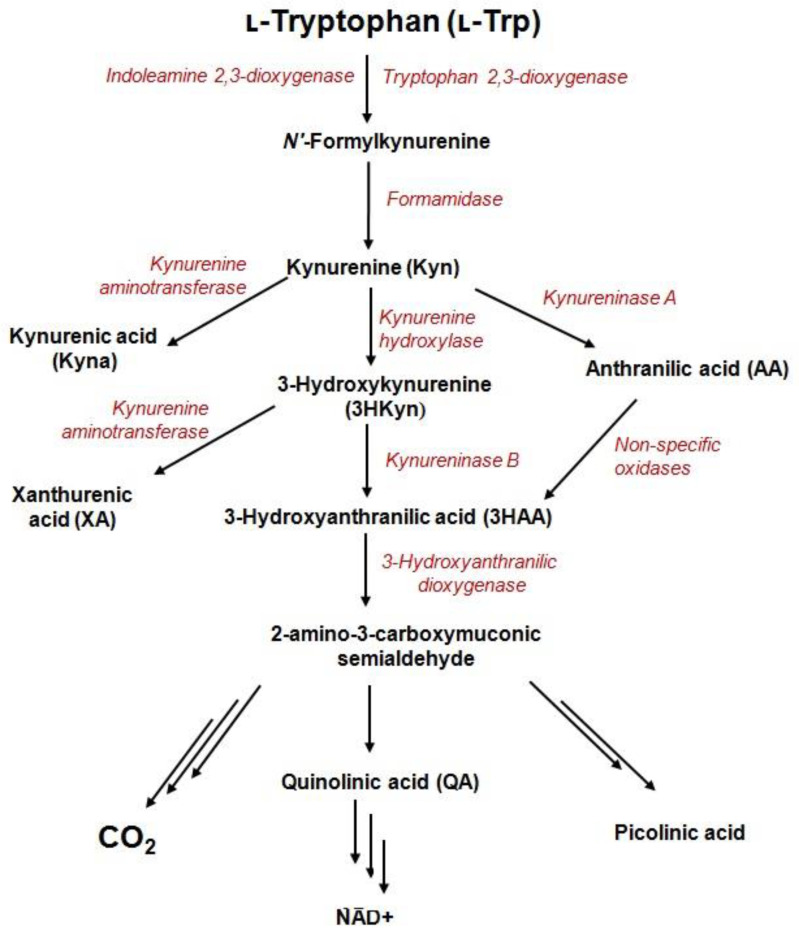
Metabolites and enzymes of the kynurenine pathway.

The kynurenine pathway (KP) is the major catabolic route of the essential amino acid, tryptophan (Trp), which generates variety of bioactive metabolites (derived from kynurenine) and an important enzyme cofactor, NAD+ (Figure 1).The activity of one of three catabolic enzymes, tryptophan 2,3-dioxygenase (TDO)—expressed in the liver—and two isoforms of indoleamine 2,3-dioxygenase (IDO1 and IDO2)—expressed in various cell types—leads to KP activation in different tissues [1,2]. During inflammation, Trp depletion via the kynurenine pathway is greatly accelerated in response to interferon-γ (IFN-γ) [3]. The process is initiated by activity of IDO1 enzyme and might take place in different cells of the human body like macrophages, dendritic, and tumor cells [3,4].KP starts from the generation of formylkynurenine, which is rapidly converted to kynurenine (Kyn)—the precursor for other catabolites including 3-hydroxykynurenine (3HKyn), kynurenic acid (Kyna), 3-hydroxyanthranilic acid (3HAA), anthranilic acid (AA), xanthurenic acid (XA), and quinolinic acid(QA) [5]. Some Trp metabolites bind to receptors expressed by immune cells to promote tolerogenic responses [4]. For instance, Kyn is an immunomodulatory molecule that inhibits T-cell proliferation, reduces the activity of natural killer cells and dendritic cells, and promotes the differentiation of regulatory T-cells (Tregs) [6,7]. Kyna promotes monocyte extravasation and controls cytokine release [6]. Furthermore, Kyn and Kyna are both ligands of aryl hydrocarbon receptor (AhR), which is involved in multiple physiological functions, tumor invasion and/or migration [8,9]. Kyna also acts as an antagonist of a glutamate receptor and the alpha-7-nicotinic acetylcholine receptor, as well as an agonist of G-protein coupled GPR35 receptor [10]. 3HAA induces apoptosis in monocyte/macrophage cell lines [11]; 3HKyn suppresses CD4+ T-cell proliferation, induces Tregs development, and prolongs corneal allograft survival [12]; and QA exerts neurotoxic effects via at least nine different mechanisms [13]. Photochemically active XA leads to apoptosis of the epithelial cells in lenses [14] and shows diabetogenic properties [5]. On the other hand, KP metabolites are recognized for their beneficial properties such as antioxidant (3HKyn, 3HAA, AA, XA, and Kyna), [5,15,16,17], neuroprotective (Kyna) [15], vasorelaxant (XA) [17], and anti-inflammatory (Kyna) factors [5].

Within the last few years, a number of comprehensive reviews have been published on the role of KP in the regulation of biological processes [5,6,10,18,19,20,21,22,23,24,25,26]; thus, this issue is only briefly discussed here. For instance, the activation of KP is involved in the prevention of miscarriage by inhibiting the activity of mother T-cells [21]. Mounting evidence from many laboratories associates KP with tumor immune surveillance [2], neuropathology [22], autoimmune disorders [23], dermatological pathologies [24], infections [25], obesity, preeclampsia, depression and stress, suicide, schizophrenia [26], and cognitive deficits [19]. Furthermore, KP metabolites are promising diagnostic and therapeutic targets in gastric cancer [27], amyotrophic lateral sclerosis [26], major depressive disorder [28], type 2 diabetes [6], and psychiatric disorders [19].

There is an urgent need to develop accurate, easy-to-use, and reliable analytical methodologies for the quantification of biologically important molecules in a variety of samples. Electrochemical sensors have tremendous potential to be the future generation of analytical systems owing to their simple operation, low laboriousness, speed, high diversity, low cost, portability, inherent miniaturization without compromising their capabilities, and high sensitivity [29,30].This approach holds great promise for obtaining the desired analytical data faster, simpler, and cheaper than currently used biochemical and instrumental assays, e.g., based on liquid chromatography-mass spectrometry. Moreover, on-going studies indicate that electrochemical sensors can be implemented in the real-time detection of target analytes [29].

Up to now, many electrochemical sensors have been developed for the determination of bioactive molecules, toxins, and environmental pollutants [30,31,32]. Nevertheless, the potential of electroanalysis in the field of KP metabolites monitoring has not been sufficiently verified. Most available methods with electrochemical detection (ECD) of these Trp metabolites exploited both chromatographic and pre-treatment steps for reducing interference from other species in complex media. Beginning in the year 2000, the number of reports utilizing this approach decreased drastically (Table 1).Meanwhile, liquid chromatography (LC) employing detection modalities like UV absorbance, fluorescence, and mass spectrometry (MS) has attracted considerable interest for studying Trp metabolism [33].In particular, expanding evidence suggests the LC-MS-based method to be the analytical gold standard for the monitoring and determination of KP metabolites in body fluids, tissues, and cultured cells. Although many LC-based protocols for KP metabolites determination have been developed [27,33,34,35,36], the number of new analytical approaches published is constantly growing from year to year. Importantly, some KP metabolites (3HKyn, QA) have unfavorable chromatographic properties (short retention time, poor peak shape, low ionization efficiency in the MS source), making their quantitative LC-MS analysis in a rich biological matrix difficult. Although precolumn derivatization can resolve the problem [37], the use of other analytical techniques for the determination of these compounds can be beneficial. Quantification of QA using high-performance liquid chromatography with ultraviolet detector (HPLC-UV) can also be problematic owing to the short retention time and tendency to overlap with 3HKyn signal [38].Accurate analytical methodologies for these two KP metabolites’ determination are highly desirable regarding their toxicity and implication in mechanisms of various diseases. All the issues suggest that new methodological advances are still required to better understand the role of Trp metabolites in pathological states.

Recently, single reports regarding the development and application of electrochemical sensors for KP metabolites quantification in biological samples without chromatographic separation began to appear (Table 1). It seems that a new era of KP research has been initiated. These works have demonstrated that electroanalytical methods are worth considering for KP metabolites’ sensing as alternative analytical tools by highlighting their advantages against popular LC-based methodologies. However, the limited number of works on this subject indicates that this is still a detectable issue and much remains to be explored.

Here, we discuss potential difficulties and challenges during conducting electrochemical measurements of KP metabolites in samples of biological origin and summarize the achievements made in the field. Finally, we present future perspectives of using electrochemical sensors in determinations of KP metabolites.

## 2. Circulation and Occurrence of KP Metabolites in Biological Samples

Mammals cannot synthesize Trp and it must be supplied by ingested proteins. Less than 1% of ingested Trp is used for protein synthesis, and most of this amino acid is metabolized along with one of four pathways by oxidation (KP), decarboxylation (tryptamine synthesis), hydroxylation (serotonin synthesis), and transamination (indolepyruvic acid synthesis) [6,18,21]. The majority of Trp is transported by the large neutral amino acid transporters into the gut, where only a fraction is used, and the rest enters portal circulation and is subjected to a liver metabolism [6].The remaining amount of Trp, together with the products degraded in a liver, is distributed to peripheral circulation and transported to different tissues including a brain, heart, and skeletal muscle [6]. Approximately 75–95% of circulating Trp is bound to albumin [18]. However, only free Trp is available for transport across the blood–brain barrier [18] and degradation pathways [6].

More than 95% of Trp is metabolized along with KP [6]. In normal conditions, Trp depletion via KP is catalyzed by TDO—a liver enzyme—and an extrahepatic enzyme IDO contributes minimally to this process (5–10%) [5]. IDO has a wide tissue and cellular distribution [60], but TDO shows predominant expression in the liver and low expression in the brain [61]. The extrahepatic KP becomes quantitatively more significant under conditions of immune activation [21]. Kyn—the first stable KP metabolite—diffuses into the bloodstream from the tissues, and then is carried to the liver and/or kidney, where it is further depleted [62]. Kyn and 3HKyn can be transported across the blood–brain barrier by large neutral amino acids carrier, while QA, Kyna, 3HAA, and AA cross it by passive diffusion [63]. The study on mice has demonstrated that maternal Kyn can cross the placenta and increase the levels of both Kyna and 3HKyn in the fetal brain, but peripherally applied Kyna does not cross the placenta [64]. Furthermore, KP metabolites like Kyna can be easily absorbed from the lumen of the digestive system [10].

As summarized in Table 2, KP metabolites are frequently found in biofluids, tissues, and cell-delivered material at low nanomolar or low micromolar concentration levels. In rat peripheral tissues (kidney, liver, lung, intestine, spleen, muscles), Kyn is present in rather lower amounts comparing to other KP metabolites in the same body fluids and tissues (Table 2). The brain tissue contains higher amount of Kyn (Table 2). In serum, plasma, peritoneal, and cerebrospinal fluids Kyna, AA, and XA contents are usually lower than other metabolites. Under physiological conditions, Kyn is preferentially converted into 3HKyn and then 3HAA and QA culminating in the generation of NAD+ [6]. The competing arms of the KP—resulting in Kyna, AA, and XA generation—are controlled by the availability of exogeneous niacin, kynurenine, inhibition of kynurenine hydroxylase, and activation of kynureninase A [5]. Among biofluids, urine is distinguished by a high content of KP metabolites, because renal excretion is the main route of elimination of Kyn and its downstream products [65]. Furthermore, the kidney can uptake Kyn and 3HKyn from the blood. These metabolites are further metabolized and excreted in the form of Kyna and XA, respectively [65]. Urinary excretion of some kynurenines (e.g., Kyna, 3HKyn, XA, and 3HAA) can increase in different conditions, such as after Trp intake [66] or lipopolysaccharide (LPS) exposure [62], and in patients with diabetes [5] or porphyria [67].

In disease conditions, abnormal Trp metabolism can be accompanied by changes in levels of KP metabolites. For instance, QA levels can be suspected to increase in plasma, cerebrospinal fluid (CSF), and/or brain in many neurological (e.g., epilepsy, Huntington disease) and inflammatory (e.g., HIV infection) conditions [5]. Patients with primary Sjögren’s syndrome have higher serum Kyn concentration than the control group [57]. In the brain, increased Kyna concentration was observed in postmortem Alzheimer’s dementia [72], similar to 3HKyn modified proteins [77]. In rats with renal insufficiency, an increase in the plasma concentration and tissue contents of KP metabolites in the kidney, liver, lung, intestine, spleen and muscles compared with healthy control has been noted [65].

KP metabolites might be also monitored in cells and cell culture medium (Table 2). Kyn is secreted at micromolar amounts into culture medium by various cell types (e.g., cancer cells, monocytes, macrophages, glioma cells, and dendritic cells), while other metabolites are usually not detected [57,75,77]. However, under some conditions such as stimulation with IFN-γ, Trp depletion is accelerated and other downstream KP metabolites are secreted (e.g., 3HKyn, 3HAA, and AA) [56,75,77]. Notably, after IFN-γ treatment, the intracellular concentrations of KP metabolites can be higher than secreted into a culture medium [74].

## 3. Analytical Methods Used for Determination of KP Metabolites

Numerous liquid chromatography-based methods have been already developed for quantification of KP metabolites in biological samples [33]. Followed a chromatographic separation, KP metabolites can be determined using an ultraviolet (UV) detector at a wavelength of 220 nm [38]. However, owing to the high impact of endogenous compounds, other wavelengths are favored in clinical analysis [33]. Fluorescence detectors (FLDs) are more selective comparing to UV ones. The main drawback of fluorometric detection is that KP metabolites generally show low or negligible native fluorescence [33,38], and derivatization to form fluorescent adducts (e.g., in reaction of a benzofurazan-type reagent with Kyn [78]) is necessary. The potential of FLD with respect to KP metabolites’ determination has been highlighted for Kyna, since the chelation with zinc ions significantly enhances the Kyna fluorescence response [79]. This strategy allows for the determination of trace levels of Kyna in plasma (Table 3).However, the measurement conditions (pH) should be carefully adjusted, due to clotting risk or interference with the formation of the chelate complex [79]. The superiority of fluorometric detection over UV has also been reported for 3HAA and AA [38].The results collected in Table 3 suggest that FLD is improper for 3HKyn and QA detection. In this case, especially at low concentrations of 3HKyn, chromatographic separation by the electrochemical detector (ECD) can be helpful, as demonstrated in Table 1. Quantitative analysis of KP metabolites performed on a LC system working with electrochemical detector (ECD) was discussed in the next section.

Liquid chromatography–electrospray ionization–tandem mass spectrometry (LC-ESI-MS/MS) allows for monitoring and quantification of a whole panel of KP metabolites simultaneously with compounds from other metabolic routes during a single run, and in a relatively short analysis time [34]. The analysis can be performed in positive and negative ion scan modes; however, the negative mode offers lower LODs for 3HAA and AA [27]. Electrospray ionization (ESI) is prone to so-called matrix effects (MEs) caused, e.g., by co-eluting matrix components, and leads to ion suppression or ion enhancement [80]. MEs are considered as a “weak” point of LC-MS-based protocols, because they affect the accuracy, precision, and sensitivity of the method, and are observed during KP metabolites’ determination in biological samples [27,34]. Matrix-matched calibration and using internal standards are popular strategies for compensation of the matrix-dependent effects effecting the analyte signal and correcting the loss of a target compound during a sample preparation step.

Gas chromatography-mass spectrometry-based methods (GC-MS) give promise for the determination of QA at lower concentration levels compared with LC-MS methodologies (Table 3). The analysis, however, requires a derivatization step. Although QA determination as dihexafluoroisopropyl, pentafluorobenzyl [81], or hexafluoroisopropyl [82] esters yields a significant increase in QA response, the applied derivatization procedures are not suitable for other KP metabolites [81]. Besides the great sensitivity and selectivity of LC-MS and GC-MS approaches, they require time-consuming sample preparation steps, using costly equipment and isotope-labeled internal standards for calibration. Utility of capillary electrochromatography-tandem mass spectrometry (CEC-MS/MS) using acrylamido-2-methyl-1-propanesulfonic acid functionalized stationary phase for determination of the endogenous concentration of Kyn, Kyna, AA, and QA in human plasma has also been demonstrated [83], but the calculated LODs are not impressive (Table 3).

**Table 3:** Analytical methods used for kynurenine pathway metabolites’ determination in biological samples (excluding methods with electrochemical detection).

Method	Biological Matrix	LOD [nM]							Ref.
		Kyn	Kyna	3HKyn	3HAA	AA	XA	QA	
HPLC-FLD	Plasma	30.00	0.09	-	-	-	-	-	[79]
HPLC-FLD	Brain	-	-	-	1.00	1.00	-	-	[84]
HPLC-FLD	Plasma, serum, liver	-	26.00	-	32.00	7.00	-	-	[38]
HPLC-UV	Plasma, serum, liver	24.00	53.00	22.00	65.00	72.00	49.00	-	[38]
UHPLC-ESI-MS/MS	Plasma	>11.52	<16.69	69.58	25.43	13.85	<5.85	<146.00	[34]
UHPLC-ESI-MS/MS	Urine	<11.52	0.53	<34.79	<203.43	13.85	<9.26	233.37	[34]
UHPLC-ESI-MS/MS	Peritoneal Fluid	1.58	1.76	7.59	10.77	12.03	4.70	5.92	[27]
GC-ECNI-MS	Cell culture medium	0.55	0.20	-	-	0.11	1.07	0.07	[81]
GC-ECNI-MS	Cerebrospinal fluid	-	-	-	-	-	-	1.00	[82]
CEC-MS/MS	Plasma	250.00	50.00	-	-	50.00	-	50.00	[83]
NCQDs-QPRTase	Serum	-	-	-	-	-	-	651.00	[85]
Fluorometry	Urine	-	n.c.	-	-	-	-	-	[86]
Spectrophotometry	Urine	-	-	-	-	-	48,740.31	-	[87]
ELISA	Plasma, serum, urine, cell culture supernatants	≥40.00	≥3.17	-	-	-	-	≥35.90	[*]

*—based on products information available at website (www.immusmol.com - accessed date 25 September 2021); CEC-MS/MS—capillary electrochromatography-tandem mass spectrometry; ELISA—enzyme-linked immunosorbent assay; GC-ECNI-MS—gas chromatography–electron capture negative ion–mass spectrometry; HPLC-FLD—high performance liquid chromatography-fluorescence detection; HPLC-UV—high performance liquid chromatography–ultraviolet detection; n.c.—not calculated; NCQDs—QPRTase -nitrogen doped carbon quantum dots immobilized with quinolinate phoshphoribosyl transferase; UHPLC-ESI-MS/MS—ultra-high performance liquid chromatography–electrospray ionization–tandem mass spectrometry.

Despite chromatography-based methods, other analytical techniques allow for quantitative analysis of KP metabolites in biological samples. A fluorescent bioprobe based on nitrogen doped carbon quantum dots immobilized with quinolinate phoshphoribosyl transferase has been designed for QA determination in serum [85], but other analytical approaches offer much better LODs (Table 3). Urinary Kyna can be determined by fluorometry after reaction with hydrogen peroxide on irradiation with UV light [86], while urinary XA can be measured by spectrophotometric procedure in the presence of Fe(III)-2,4,6-tris-(pyridyl)-s-triazine complex in acidic buffer [87]. Besides the simplicity of the above protocols, the obtained LODs are much higher than for other analytical methods (Table 3). Enzyme-linked immunosorbent assay (ELISA) combined with reaction of the analyte and Ehrlich reagent can be useful for Kyn determination in cells’ supernatants [88]. Quantitation is carried out by the measurement of absorbance at a specific wavelength. ELISA kits for Kyn, Kyna, and QA are commercially available and designed for working with small amounts (~20–50 µL) of plasma, serum, urine, and cell culture supernatants on the multi-well plates. The sensitivity of ELISA varies depending on analyte, biological matrix, and manufacturer, which is exemplified in Table 3. Despite the high flexibility of ELISA, the assay is relatively expensive as each analyte requires a specific antibody, which, in some cases, presents limited reactivity.

## 4. Electrochemical Detection of KP Metabolites after Chromatographic Separation

Liquid chromatographic systems coupled with electrochemical detectors (LC-ECDs) were used for KP metabolites’ determination in a variety of biological samples from over four decades (Table 1). KP metabolites like Kyn, Kyna, 3HKyn, 3HAA, AA, and XA are electroactive and can be monitored using electrochemical detectors working with bare and modified carbon-based electrodes or coulometric electrode array system. These methods were preferably used for the study of Trp metabolism via KP in brain tissue, but the application for other biological matrices was also reported (Table 1). It should be highlighted here that LC-ECD methods allow for 3HKyn and 3HAA determination at concentration levels lower compared to HPLC-UV and even UHPLC-ESI-MS/MS (Table 1 and Table 3).

Nevertheless, these approaches require sample pre-treatment step and chromatographic separation to reduce interference from electroactive species present in the complex media. Furthermore, they suffer from poor reproducibility mainly due to an electrode fouling, and other modern detectors allow for the measurement of a broader number of analytes and with better sensitivity [33]. Importantly, there are other analytical issues, i.e., difficulty of direct QA measuring with conventional working electrodes or capability of Kyna detection at physiologically relevant levels associated with high oxidation potential [54]. The problems with methodology are discussed in the next paragraph. As result, a significant decrease in the usage of electrochemical detectors in conjunction with liquid chromatography systems is observed, as presented in Table 1. Certainly, the introduction of mass spectrometers to laboratories brought new opportunities in the field of analytical chemistry and contributed to the development of LC-ECD methods.

## 5. Developments in Electrochemical Sensors for the Determination of KP Metabolites

The basis of the electrochemical analysis is the reaction that occurs on the surface of the working electrode. Thus, the selection of the working electrode is the crucial preliminary step for successful electrochemical analysis [89]. Oxidation signals of KP metabolites (Kyn, Kyna, 3HKyn, 3HAA, AA, and XA) can be measured at a bare glassy carbon electrode (GCE) by differential pulse voltammetry (DPV). Glassy carbon is widely used as an electrode material because of its chemical stability, broad potential window, and low cost [89]. Table 4 collects parameters on the oxidation potentials (E_ox_) of different KP metabolites estimated in phosphate buffered saline (PBS) at pH 7.7. Under experimental conditions, QA signal was not detected. One can assume that the QA oxidation peak appears at a high oxidation potential and is difficult to measure at a bare GCE. Owing to high E_ox_, electrochemical determination of Kyna can be also problematic. Such an assumption can be supported by the data obtained by Kato et al., who calculated the theoretical electrochemical E_ox_ of QA and Kyna at nanocarbon film electrode to be equal 2.36 V and 1.40 V, respectively (50 mM phosphate buffer pH 7.0 as the supporting electrolyte) [40]. To note, in DPV at GCE in typical supporting electrolytes for potentials >+1.2 V, the background current increases and baseline drift is observed. It significantly decreases the S/N and makes it difficult to measure signals from analytes. A boron-doped diamond electrode (BDDE) exhibits wider electrochemical windows for aqueous media [90] and a lower background current magnitude than GCE [42], making BDDE more suitable for Kyna detection. Nanocarbon film electrode with a high sp^3^ content exhibits a higher S/N ratio against Kyna compared with GCE or BDDE, but estimated LOD in phosphate buffer pH 7.0 (0.4 µM) [40] is not sufficient to detect this metabolite in many biological matrices (Table 2). This electrode can also be applied for quantification of other KP metabolites (with QA being an exception) [40], but LODs are not impressive either (Table 1). All the above suggest that new electrode materials with high electrocatalytic activity or other solutions should be exploited to allow direct electrochemical detection of QA and Kyna at low physiologically relevant levels. Addressing these limitations, QA and Kyna are currently preferably measured in biological samples by LC-MS-based methods [27,54,70]. It is noteworthy that QA is difficult to quantify by LC-MS or LC-MS/MS because of short retention time, low *m/z*, and poor ionization efficiency in the MS source [37]. The determination of this metabolite using HPLC-UV can also be problematic [38].

So far, only one paper reports indirect electrochemical detection of QA in human serum using a biosensor modified with quinolinate phosphoribosyl transferase (QPRT) enzyme [46]. In this approach of the sensor design, glass support coated with indium tin oxide (ITO) has been modified with reduced graphene oxide and QPRT enzyme. The formation of the enzyme-ligand complex (QA-QPRT) at the electrode surface leads to electron release that is monitored through DPV. This method exhibited linearity in the concentration range from 6.5 μM to 65 mM, and was tested in a diluted serum matrix. However, QA concentration in serum is frequently <1.0 μM (Table 2) and, together with a need for sample dilution before analysis, represents a limitation for the applicability of this sensor. Admittedly, dilution of the sample minimizes the negative influence of the matrix components on the sensitivity, however the concentration of the analyte also decreases. Furthermore, other analytical methodologies offer lower LODs in QA determination (Table 3). Regarding QA in different tissues and biological fluids (Table 2), further improvements should be worked out to design the electrochemical sensor capable of 0.01 μM concentration or lower in the presence of the sample matrix. Because QA exerts multiple neurotoxic effects [13] and difficulties in determination by LC-MS- and HPLC-UV-based methods [37,38], the selective and sensitive sensor represents an attractive analytical tool to satisfy the growing demand for rapid and reliable quantification of this toxin in biological material.

Direct simultaneous determination of Kyna and XA [44] or 3HAA and AA [16] at a bare GCE using DPV has already been applied to study the antioxidant activities of their coordination complexes with Fe. These metabolites display good resolution of their voltammetric signals and can be measured simultaneously (Table 4). However, the study was conducted only in phosphate buffer (0.1 M, pH 7.4) in the absence of a biological matrix, and LODs were not calculated. Thus, it is difficult to assess the usefulness of these protocols for quantitative analysis of Kyna, XA, 3HAA, and AA in biological samples.

It seems that bare electrodes are not an ideal choice to conduct direct and selective electrochemical determinations of KP metabolites at low physiological concentration levels. Furthermore, considering the relatively high E_ox_ of Kyna at bare carbon-based electrodes and possible interferences from sample matrix components, electrochemical determination of Kyna can be difficult or even impossible. Modification/functionalization of the electrode surface is a well-recognized and effective strategy for the improvement of both sensitivity and selectivity of electrochemical measurements [30], and can be useful in the quantification of Kyna and other KP metabolites. One example is the functionalization of the multi-electrode platform composed of a BSA-pseudo Kyna molecule that, in combination with the selective Kyna antibody and subsequent interaction with the anti-IgG-HRP antibody (secondary-Ab), was used to fabricate a sensor. It allowed for Kyna determination at low nM concentrations by chronoamperometry or electrochemical impedance spectroscopy [39]. It is currently the most sensitive electrochemical sensor developed for Kyna quantification in biological samples. It offers lower or similar LODs compared with those reported for HPLC-UV and LC-MS/MS, respectively (Table 3). However, the biosensor is not inert to the sample matrix. Sample dilution is recommended to minimize nonspecific adsorption of serum components on the surface of the electrode occurs.

Advantages of the GCE and BDDE surface modification have also been emphasized by our group during the design of voltammetric sensors for Kyn [41,42]. Electrochemical deposition of Bi film onto the BDDE surface presents an easy and rapid way to improve the sensor sensitivity toward Kyn measured by DPV, and it reaches a low LOD (30 nM) [42]. The coating of the GCE surface with a thin layer of Nafion polymer allows for detection of lower contents of Kyn (Table 1), as the cationic form of this molecule can be pre-concentrated onto the electrode surface before the stripping step [41]. Nafion—perfluorinated polymer containing functional sulphonic acid end groups—is highly conductive to protons and acts as an exchanger membrane [91], causing the repulsion of anions and attraction of positively charged species. The Nafion layer can be formed by the drop-coating method (without the need for sophisticated apparatus) and easily removed by polishing using alumina slurries. Furthermore, Kyn can be effectively accumulated onto the Nafion-coated GCE at the potential of +0.5 V in 0.1 M H_2_SO_4_, before being stripped by scanning potential toward more positive values [41].This strategy allows for working with diluted samples and decreases some interferences delivered from the sample matrix components. However, these two sensors are not specific to Kyn, and their accuracy should be verified using comparative approaches with respect to the biological matrix of interest. So far, the applicability of Bi film-modified and Nafion-coated sensors was confirmed for the analysis of material derived from cultures of human cancer cells. Karami et al. have also developed the sensor for Kyn quantification in culture medium collected from cancer cells, but applying a multi-stage modification of the surface of the screen-printed gold electrodes (AuSPEs) [45]. The protocol for the modification of AuSPEs’ surface includes the deposition of carboxylated multiwall carbon nanotubes and immobilization of monoclonal antibody (mAb) specific to Kyn. The immunosensor has the capability of being integrated into lab-on-a-chip, microfluidics, and micro total analysis systems and allows simultaneous detection of Kyn and Trp. Notably, the above electrochemical sensors offer competitive LODs of Kyn with chromatographic-based approaches (Table 1 and Table 3), without the need for the time-consuming and muli-step sample preparation. While there have been a few efforts to direct electrochemical determination of Kyn in material derived from cells, there has been no study aimed at the analysis of other kinds of biological matrices, like serum.

Other researchers proposed the application of molecularly imprinted polymers (MIPs) to hold out the possibility of selective electrochemical measurements of 3HAA in urine [47]. Molecular imprinting represents an efficient method for the synthesis of materials with highly specific organization. The method is based on co-polymerization of a functional monomer and a cross-linker in the presence of a target molecule (so-called imprint molecule), which acts as a molecular template. The formation of specific binding sites between the polymer and the template can be provided via the self-assembling of the template and the functional monomer. After removal of the imprint molecule from the synthesized polymer network, the selective rebinding of the template in the sample solution can occur [92].To fabricate a sensor for 3HAA, the AuSPE was coated with a solution of poly(ethylene-co-vinylalcohol) containing the template molecule, released further by a surfactant. The sensor works with integrated potentiostat in flow injection mode and allows for 3HAA determination at low nanomolar levels [47]. Importantly, this MIP-modified sensor is an attractive analytical tool for determination of urinary 3HAA, as the obtained LOD is much better than that reported in the urine matrix by LC-MS/MS [34].

Until now, no electrochemical sensor for direct or indirect determination of 3HKyn has been developed. The available data concern only electrochemical detection of this neurotoxin in biological material after chromatographic separation (Table 1). This metabolite generates a good oxidation signal even at the unmodified GCE and can be measured using DPV (Table 4). Direct electrochemical detection of 3HKyn (without chromatographic separation) is possible, but not straightforward, as the measurement conditions need to be properly selected to ensure satisfactory selectivity (see the next section) and low LODs (at least nM) in the presence of the sample matrix.

## 6. Interferences

Measurement of Trp and its metabolites is difficult because of their lability, low physiological concentration, and presence of interfering compounds present in biological samples. Protein precipitation, purification using solid-phase extraction (SPE), concentration, and filtration, is frequently used for biological sample pre-treatment before LC-based analysis [33]. In case of electrochemical sensors, the appropriate selectivity against electrochemically active species coexisting with analytes in a sample matrix is required [32]. In this section, possible interferences during an electrochemical determination (without prior chromatographic separation) of KP metabolites in biological samples are discussed.

**Figure 2:**
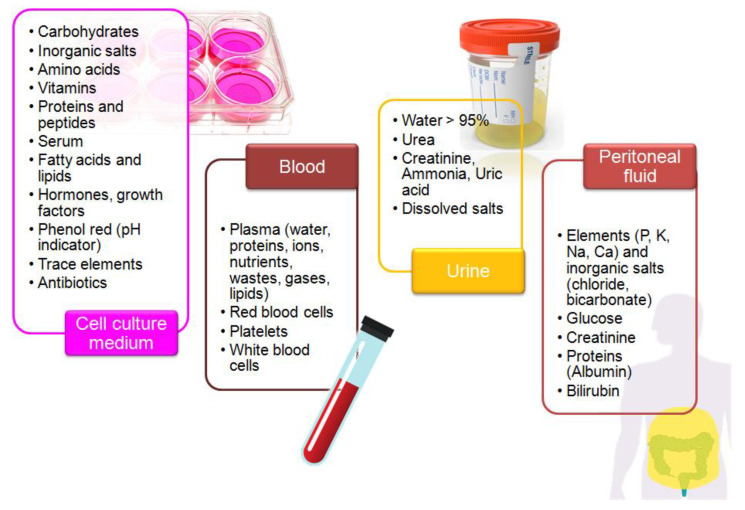
Composition of the selected biological matrices used in studies on tryptophan metabolism.

Biological samples are complex media because they contain extra organic and inorganic components, along with the presence of trace amounts of a target molecule [92]. Furthermore, their composition strongly differs and depends on the sample type (Figure 2). Overlapping signals from electro-active compounds that have relatively close oxidation potentials pose one of the major problems during electrochemical analysis of a real sample. The careful selection of the electrode support and measurement conditions allows for improvement of selectivity and sensitivity of electrochemical measurements [32,42]. Regarding the electrochemical determination of KP metabolites, special attention should be paid to the following issues:

- Influence of Trp on Kyn signal

Trp signal tends to overlap with Kyn peak in supporting electrolytes at pH lower than 4.6 [42]. In the analysis of samples with similar concentration of Trp and Kyn, the selectivity problem can be resolved by working in the supporting electrolyte at pH 6.5 [42] or in an acidic medium using a Nafion-coated electrode [41]. In case of biological samples containing much higher amounts of Trp in relation to Kyn, e.g., serum, further developments are required;

- Overlapping signals from 3HKyn and 3HAA

Voltammetric signals of these two KP metabolites appear at similar potentials (Table 4). The challenge with 3HKyn and 3HAA is a comparably low (nM—µM) occurrence in biological materials (Table 2) providing false results. So far, no electrochemical sensor has been designed for direct simultaneous determination of 3HKyn and 3HAA without employing chromatographic pre-treatment;

- Influence of Trp on AA signal

**Figure 3:**
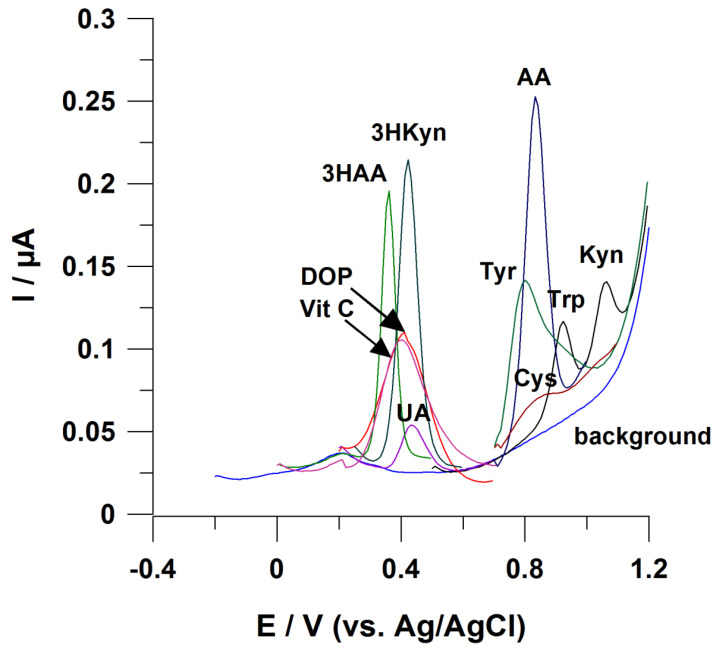
Possible interference in the case of voltammetric determination of KP metabolites. Working electrode: BDDE modified with Bi film; supporting electrolyte: 0.25 M mixture of CH_3_COONH_4_ with CH_3_COOH (pH 6.5), 0.04 M potassium sodium tartrate, and 0.5 µM Bi(III); registration technique: DPV (for details, see [42]). Concentration of analytes: 3HKyn 10 µM, 3HAA 2 µM, ascorbic acid (Vit C) 50 µM, dopamine (DOP) 20 µM, and uric acid (UA) 2 µM; tyrosine (Tyr) 20 µM, cysteine (Cys) 10 µM, tryptophan (Trp) 2 µM, kynurenine 2 µM, and anthranilic acid (AA) 10 µM.

Signals from these molecules appear close to each other at the voltammogram (Figure 3). Difficulties in AA electrochemical measurements are to be expected, while biological samples contain this metabolite at significantly lower amounts compared with Trp (Table 2). To the best of our knowledge, no electrochemical sensor working without employing chromatographic separation of sample components has been developed to address this analytical challenge;

- Interferences delivered by Trp metabolites formed in other pathways

In addition to oxidation (kynurenine pathway), Trp might undergo hydroxylation and decarboxylationor transamination [21] to yield electro-active compounds such as indoxyl acetate, indoxyl sulfate, melatonin, 5-hydroxyindoleacetic acid, and 5-hydroxytryptamine (serotonin). These bioactive molecules can be present in biological samples at lower, similar, or higher concentration levels in relation to KP metabolites, depending on the cellular conditions and pathological state. Importantly, they can influence signals of KP metabolites. For instance, an enhancement or decrease in Kyn peak current during voltammetric measurements has been reported [41];

- Effect of amino acids

As some amino acids can be directly electrooxidized on solid electrodes [93], their effect on electrochemical measurements of KP metabolites should be checked. For instance, amino acids like methionine and tyrosine can influence the voltammetric signal from Kyn [41]. Furthermore, as demonstrated in Figure 3, AA voltammetric signal is prone to interferences from tyrosine and cysteine. Some possible interferences from Trp (the substrate for generation of KP metabolites) were discussed above;

- Interferences from uric acid (UA), ascorbic acid (Vit C), and dopamine (DOP)

Figure 3 highlights a tendency of these molecules to disturb electrochemical measurements of 3HKyn and 3HAA, as they have similar E_ox_. UA, DOP and Vit C are present in body fluids (e.g., urine, serum) [94,95,96], making these interferences likely to occur. So far, only one report explored direct electrochemical determination of 3HAA in urine [47], and the selectivity of the measurement was provided by the application of molecularly imprinted polymer (MIP) as modifiers of a working electrode surface. Furthermore, UA, Vit C, and DOP can interfere with a Kyn voltammetric response that was previously described [41,42].

In case of electrochemical measurements of KP metabolites, the following strategies can be useful for elimination or minimalization of interferences delivered from the components of the biological milieu:

- Dilution of the sample;
- Sample pre-treatment by SPE

The strategy can be useful for the elimination of impurities and interfering compounds, analyte isolation and/or pre-concentration. Sample clean-up using SPE is based on partitioning of analytes between the liquid phase and the solid sorbent. In recent years, new solid sorbents for SPE to facilitate the extraction procedure in complicated matrices have been developed. To this end, sorbents such as metal organic frameworks (MOFs), MIPs, carbon nanotubes, magnetic nanoparticles, graphene and graphene oxide, and metallic nanoparticles have been proposed for the separation, concentration, and determination of different analytes in biological samples [92]. The utility of these novel SPE sorbents for trace analysis of KP metabolites is worth investigation;

- Protein removal

Protein adsorption blocks the electrode surface, reducing its effective area and impacting charge transfer across the electrode–solution interface [97]. Protein fouling on platinum electrode [97], GCE [98], and gold electrode [99] has been reported. The problem can arise during the analysis of protein-rich samples like serum or measurements using implantable electrodes. Using bovine serum albumin (BSA) as a model protein, showed that mass adsorption is greater when the gold electrode surface and the protein have the opposite charge [99]. Modification of the electrode surface with hydrophilic groups reduced protein adsorption and may be useful for the analysis of clinical samples [98]. Utility of a simple protein precipitation from the sample before electrochemical analysis using trichloroacetic acid, hydrochloric acid, acetonitrile, methanol, or acetone [33] can be also considered;

- Limiting access of the interfering compounds to the electrode surface

Covering the working electrode surface with perm-selective membranes can limit access of interfering species to the reactive surface of the sensor. Membranes such as Nafion (anionic polymer, to exclude an effect of negatively charged species such as ascorbate and nitrite), chitosan (a natural biopolymer, to exclude interferences from ascorbic acid), electropolymerized layers of pyrrole, polyphenylenediamine, poly-L-lysine, and poly-4-styrenesulfonate (to eliminate interferences through molecular sieving by modulating pore dimensions) can be taken into consideration [32];

- Modification/functionalization of the working electrode surface to improve selectivity towards KP metabolites

So far, MIPs [47] and antibodies [39,45] have been used as modifiers of the electrode surface to improve selectivity towards 3HAA, Kyna, and Kyn. New achievements in this field are welcomed.

## 7. Comparison of Electrochemical Sensors to LC-Based Methods

Nowadays, the analytical gold standard for the determination of KP metabolites in samples of different origin involves chromatographic analysis mainly using LC-MS/MS, as it allows for monitoring the whole panel of KP metabolites during a single analysis. Our survey indicated that HPLC-UV methods have also found a broad application in study on Trp [33]. Thus, in this section, we attempt to compare the main technical aspects of working with electrochemical sensors, LC-MS/MS, and HPLC-UV systems to highlight the advantages/disadvantages of these approaches.

Based on data presented in Table 5, it can be concluded that analysis using electrochemical sensors is distinguished by simple operation, low cost, and speed compared with HPLC-UV or LC-MS/MS. Furthermore, electrochemical measurements are closer to meeting the principles of green chemistry regarding low waste production and organic solvent consumption. However, measurements using electrochemical sensors allow for the determination of small number of analytes using a single protocol, which is limited by the potential window and requirement for electro-activity. This is the reason that electrochemical analysis is less powerful compared with HPLC-UV and LC-MS/MS. Regarding the analysis of biological samples, a sample amount is frequently limited, and must be considered in selecting an analytical method. In general, chromatography-based approaches require lower amounts of sample for analysis. However, electroanalysis coupled with screen-printed electrodes allows for conducting an analysis using a single drop of the sample [100] and shows a great opportunity for miniaturization and the construction of all-in-one devices [101].

## 8. Future Research Directions

Two major challenges faced by electrochemical measurement of KP metabolites in biological material are sufficient selectivity and sensitivity of the analysis. Possible interferences from the electro-active compounds present in the sample matrix were discussed above. To improve the selectivity of electrochemical determinations of KP metabolites, new analytical solutions on the sample preparation step involving the isolation of the analyte or removal of interfering compounds from the sample matrix before the measurement should be developed. Furthermore, the elimination of the necessity to employ chromatographic system to ensure the acceptable selectivity of the measurement will be beneficial. Next, achievements in the field of development of new electrode substrates showing wide potential window are required to make possible the direct electrochemical determination of QA. Meanwhile, different strategies of modification/functionalization of the working electrode surface should be tested to allow selective detection of KP metabolites at nM and lower concentration levels in the complex biological matrix. The sensitivity of electrochemical measurements at bare carbon-based electrodes can be insufficient for monitoring of KP metabolites at physiologically relevant levels, thus the application of the state-of-the-art nanostructured materials (nanorods, nanocubes, nanoneedles, nanoflowers, nanotubes, nanobundles [31]) as modifiers of the electrode surface is worth considering. Owing to the high reactive surface area, high conductivity, and large surface-to-volume ratio [30], nanomaterials have a great potential for the improvement of both sensitivity and selectivity of the electrochemical sensors towards KP metabolites.

Further development of electrochemical methods for monitoring and quantification of KP metabolites in a variety of biological milieus could fit in the current directions of modern analytical chemistry. New developments in the following areas are extremely important:

- Miniaturization and development of ultra-small electrodes (ultramicroelectrodes) for monitoring of KP metabolites directly in the living tissue in vivo or from a single biological cell in vitro.

Electroanalysis offers tremendous promise for scaling down analytical systems [29]. Ultramicroelectrodes, with dimensions in the range of micrometers, be inserted into the tissue of a living animal or in a large single cell immobilized in a culture plate [102]. Fast voltammetry can be used to monitor the local changes in concentrations of KP metabolites in, e.g., brain tissue related to physiological processes such as inflammation. Ultramicroelectrodes are promising tools that can allow for the identification and quantification of molecules secreted from the living cell, and to study the secretion kinetics and mechanisms;

- Development of sensors delivering reliable measurements within a few minutes of analysis period, using blood sample from a finger prick without additional pretreatment steps.

The whole blood represents an attractive material for clinical research thanks to its ready availability, simplicity, and minimally invasive method of collection, and allows to follow dynamic changes in cellular information on molecular clues of infection, inflammation, and autoimmune diseases;

- Development of portable electrochemical analyzers for a near-patient clinical blood or urine testing.

As KP metabolites hold great promise as biomarkers of many human diseases, portable analyzers allowing for their rapid and on-side determination without sample transfer to the centralized laboratories can ignite tremendous interest in the near future;

- Design and fabrication of wearable electrochemical sensors for non-invasive, continuous real-time monitoring of tracking dynamic changes in concentrations of KP metabolites within body fluids, e.g., after drug treatment.

Wearable chemical sensors can be useful in analysis of easily accessed biofluids such as interstitial fluid, sweat, saliva, and tears, and provide new insights into KP association with disease states. So far, some developments in the field of fabrication of wearable electrochemical device in the form of a flexible tattoo, microneedles, and gloves for on-body monitoring and detection of alcohol, nicotine, caffeine, DOP, and drugs have been observed and reviewed recently [103].

**Figure 4:**
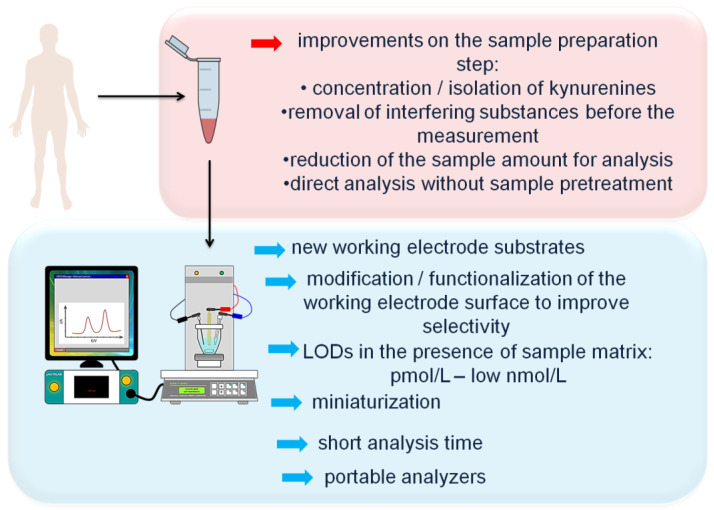
Future research direction in the field of electrochemical determination of kynurenine pathway metabolites in biological samples.

The main future research directions in the field of development of electrochemical methodologies for determination of KP metabolites are presented in Figure 4.

## 9. Conclusions

Exploration of the role of tryptophan metabolism provides novel diagnostic and treatment opportunities; however, it requires reliable methods for quantification of its metabolites in a variety of biological samples. In this review article, we summarized the achievements made in the field of electrochemical detection of tryptophan metabolites from the kynurenine pathway (KP metabolites) in biological matrices. Despite little achievements in this field, electrochemical sensors have promise of being attractive analytical tools for the reliable determination of KP metabolites at physiologically relevant concentration levels. The examples provided show the scope, power, and challenges of electrochemical measurements of KP metabolites. Nevertheless, new progress toward designing electrochemical sensors and other improvements on the measurement or sample preparation steps should be continued.

This review will be helpful to open new ideas and prompt to explore the potential of the electrochemical sensors for accurate, precise, sensitive, and selective detection of kynurenine pathway metabolites.

## Figures and Tables

**Table 1:** Progress and achievements in electrochemical determination of kynurenine pathway metabolites.

Method	Working Electrode	LOD [nM]							Application	Year	Ref.
		Kyn	Kyna	3HKyn	3HAA	AA	XA	QA			
CA	anti-IgG-HRP-MUA/MU-AuEs	–	0.02 a 0.39 b	–	–	–	–	–	Serum	2021	[39]
EIS	anti-IgG-HRP-MUA/MU-AuEs	–	0.04 a 0.28 b	–	–	–	–	–	Serum	2021	[39]
SWV	NCE (sp^3^ = 16%)	3800.00	–	14,000.00	–	10,000.00	–	–	–	2021	[40]
	NCE (sp^3^ = 47%)	1600.00	400.00	200.00	400.00	2400.00	–	–			
DPAdSV	Nafion/GCE	5.10 (60 s) *	–	–	–	–	–	–	Cellular lysate Culturing medium from cancer cells	2021	[41]
		0.59 (600 s) *	–	–	–	–	–	–			
DPV	Bi/BDDE	30.00	–	–	–	–	–	–	Culturing medium from cancer cells	2020	[42]
HPLC-ECD	dGCE	36.77	–	–	–	30.61	10.96	–	Hippocampus and ileum tissues Blood	2019	[43]
DPV	GCE	–	n.c.	–	–	–	n.c.	–	Phosphate buffer	2019	[44]
CC-PSA	mAb-MWCNT -AuSPE	0.50	–	–	–	–	–	–	Culturing medium from cancer cells	2017	[45]
DPV	QPRT-BSA -RGO-ITO	–	–	–	–	–	–	6500.00	Serum	2017	[46]
AMP	MIPs/SPE	–	–	–	19.98	–	–	–	Urine	2015	[47]
DPV	GCE	–	–	–	n.c.	n.c.	–	–	Phosphate buffer	2015	[16]
HPLC-ECD	NCE	–	0.20 **	–	–	–	–	–	Culturing medium fromastrocytes	2012	[48]
HPLC-ECD	MWCNT/GCE	500.00	–	–	–	–	–	–	Plasma	2011	[49]
HPLC-ECD	–	–	–	3.00	3.00	–	2.00	–	Plasma	2006	[50]
HPLC-ECD	–	6.25	27.51	5.58	4.90	14.23	12.20	–	Brain tissue	2002	[51]
HPLC-ECD	GCE	–	–	n.c.	–	–	n.c.	–	Mosquito larval	1998	[52]
HPLC-ECD	PGE	–	–	n.c.	–	–	–	–	Phosphate buffer	1997	[53]
CEEC	CFE	3.10	22.20	0.40	6.00	3.30	0.60	-	Brain tissue	1995	[54]
HPLC-ECD	–	–	–	n.c.	–	–	–	–	Brain tissue	1992	[55]
HPLC-CEAS	–	n.c.	n.c.	n.c	–	–	–	–	Brain tissue	1992	[56]
HPLC-ECD	GCE	–	–	n.c.	n.c.	–	–	–	Brain tissue	1991	[57]
HPLC-CEAS	–	–	n.c.	–	–	–	–	–	Brain tissue	1990	[58]
HPLC-ECD	GCE	–	–	8.75	–	–	–	–	Brain tissue	1988	[59]

*—accumulation time; **—limit of quantification; a—in buffer; b—in sample matrix; AMP—amperometry; anti-IgG-HRP-MUA/MU-AuEs— platform of 5 gold electrodes modified with self-assembled monolayer of 11-Mercaptoundecanoic acid (MUA) and 11-Mercapto-1-undecanol (MU), BSA-pseudo-Kyna, primary and secondary antibodies specific to Kyna; BiF/BDDE—boron-doped diamond electrode modified with bismuth nanoparticles; CA—chronoamperometry; CC-PSA—constant current-potentiometric striping analysis; dGCE—dual glassy carbon electrode; DPAdSV—differential pulse adsorptive stripping voltammetry; CEEC—capillary electrophoresis with electrochemical detection; CFE—carbon fiber electrode; DPV—differential pulse voltammetry; EIS—electrochemical impedance spectroscopy; GCE—glassy carbon electrode; HPLC-CEAS—high performance liquid chromatography with a 16-sensor coulometric electrode array system; HPLC-ECD—high performance liquid chromatography with electrochemical detection; mAb-MWCNT-AuSPE—gold screen-printed electrode modified with carboxylated multiwall carbon nanotubes and monoclonal antibody; MIPs/SPE—screen-printed gold electrode modified with 3HAA-imprinted polymer; MWCNT/GCE—multi-wall carbon nanotube-modified glassy carbon electrode; Nafion/GCE—Nafion film modified glassy carbon electrode; NCE—nanocarbon film electrode; SWV—square wave voltammetry; PGE—porous graphite electrode; QPRT-BSA-RGO-ITO—quinolinate phosphoribosyl transferase enzyme-reduced graphene oxide—indium tin oxide coated glass plate blocked with BSA.

**Table 2:** Comparison of concentrations levels of tryptophan and kynurenine pathway metabolites in biological samples.

Biological Matrix		Concentration Range [µmol/L or µmol/kg]							Ref.
	Trp	Kyn	Kyna	3HKyn	3HAA	AA	XA	QA	
Human serum *	41.54–95.60	1.12–2.41	0.01–0.09	0.04–0.26	0.17–0.67	4.70–12.10a	0.02–0.06	0.37–0.60	[68]
Human serum from gastric cancer patients	5.15–109.56	0.20–1.41	0.01–0.12	0.02–1.80	-	-	0.03–0.27	0.07–0.15	[27]
Human serum from amyotrophic lateral sclerosis patients	137.70–148.90	3.80–4.20	-	-	-	-	-	0.35–0.39	[26]
Horse plasma	42.50–129.74	0.41–12.69	0.11–0.15	0.69–1.90	-	-	0.21–0.46	-	[69]
Rat plasma *	35.90–42.30	1.50–1.70	0.04–0.05	0.04–0.06	-	0.04–0.06	0.03–0.04	0.30–0.41	[65]
Human peritoneal fluid from gastric cancer patients	0.34–11.22	0.04–4.06	2.52–21.04 a	0.02–3.20	0.16–2.03	0.02–0.43	0.02–0.16	0.01–1.75	[27]
Human cerebrospinal fluid *	1.70–2.72	0.03–0.09	0.80–4.00 a	1.80–7.60 a	<1.00 a	2.60–6.80a	0.30–2.30 a	0.02–0.08	[68]
Human cerebrospinal fluid from amyotrophic lateral sclerosis patients	4.80–5.20	0.21–0.25	-	-	-	-	-	~0.05	[26]
Human urine *	6.37–25.00	2.40–9.13	23.18–45.50	28.44–47.11	35.29–53.59	1.46–14.60	25.85–33.66	-	[70]
Human urine from bladder cancer patients	109.94–167.68	9.11–12.42	13.91–31.96	36.57–51.74	54.95–61.89	61.97–219.22	17.95–30.19	-	[70]
Human salvia *	0.17–0.27	0.28–0.35	2.60–3.60 a	4.80–6.20 a	3.00–3.60 a	-	-	-	[71]
Human hippocampus *	-	10.10–13.70 b	2.32–3.44 b	4.52–6.02 b	-	-	-	-	[72]
Human hippocampus from vascular encephalopathy patients	-	12.85–13.67 b	3.91–5.15 b	8.32–9.72 b	-	-	-	-	[72]
Human temporal cortex *	-	-	-	40.14–539.66	-	-	-	-	[55]
Human temporal cortex from Huntington’s disease patients	-	-	-	280.98–700.22	-	-	-	-	[55]
Human temporal cortex from Alzheimer’s disease patients	-	-	-	182.86–548.58	-	-	-	-	[55]
Mice cerebral Cortex *	120.07–127.39	~0.18	~0.07	~0.03	-	-	-	-	[73]
Mice hippocampus *	137.05–201.97	~0.30	~0.12	~0.06	-	-	-	-	[73]
Rat kidney *	~0.10	2.80–3.20 a	0.75–0.89	0.28–0.33	-	0.11–0.16	0.40–0.46	0.19–0.26	[65]
Rat liver *	0.04–0.05	5.70–6.70 a	0.15–0.18	0.53–0.59	-	0.12–0.15	0.12–0.15	0.33–0.41	[65]
Rat lung *	0.02–0.03	4.70–5.30 a	0.16–0.18	0.06–0.13	-	0.03–0.04	0.05–0.07	0.27–0.38	[65]
Rat intestine *	0.08–0.10	4.30–4.70 a	0.08–0.10	0.03–0.05	-	0.07–0.09	0.04–0.05	0.70–0.92	[65]
Rat spleen *	0.04–0.05	3.00–3.20 a	0.12–0.14	0.21–0.26	-	0.12–0.14	0.07–0.09	0.35–0.55	[65]
Rat muscle *	~0.01	5.90–6.70 a	0.18–0.21	0.05–0.06	-	0.14–0.17	0.16–0.19	0.07–0.15	[65]
Medium from human ovary SK-OV-3 cancer cells culture	-	0.69–4.93	-	-	-	-	-	-	[41]
Medium from human brain LN-229 cells culture **	n.d.	31.90–32.50	-	~0.10	0.70–0.90	-	-	-	[74]
Human brain LN-229 cells **	12.00–16.80	308.50–380.50	-	1.40–1.80	1.20–1.40	-	-	-	[74]
Medium from human endometrial cancer cells culture	-	1.22–12.26	-	-	-	-	-	-	[75]
Medium from human peripheral blood mononuclear cells culture	91.10–101.50	0.24–0.32	-	-	<0.01	-	-	-	[76]

a—values in nmol/L or nmol/kg; b—values in pmol/mg protein; *—collected from healthy animals; **—stimulated with IFN-γ; n.d.—not detected.

**Table 4:** Physicochemical properties and oxidation potentials of kynurenine pathway metabolites determined by DPV on GCE in 0.1 M PBS (pH 7.7).

Metabolite	Chemical Formula	Structure	E_ox_ * [V] (pH = 7.7)	Isoelectric Point [37]
Kyn	C_10_H_12_N_2_O_3_	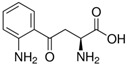	0.86	6.11
Kyna	C_10_H_7_NO_3_	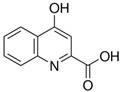	1.06	2.31
3HKyn	C_10_H_12_N_2_O_4_	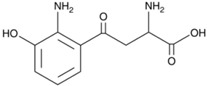	0.42	6.11
3HAA	C_7_H_7_NO_3_	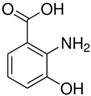	0.36	3.03
AA	C_7_H_7_NO_2_	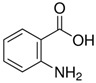	0.76	3.34
XA	C_10_H_7_NO_4_	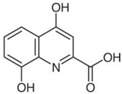	0.65	3.25
QA	C_7_H_5_NO_4_	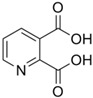	n.d.	2.27

*—estimated in three-electrode cell consisted of GCE (d = 1 mm, working electrode), Pt wire (counter electrode), and Ag/AgCl (reference electrode);concentration of the analyte: 50 µM; the scan potential:100 mV; n.d.—not detected.

**Table 5:** Comparison of technical aspects of routine work with electrochemical sensors, LC-MS/MS, and HPLC-UV systems.

Feature	Analysis Using Electrochemical Sensors	HPLC-UV	LC-MS/MS
Setup	System consisting of potentiostat/galvanostat, stand, electrochemical cell, and a computer equipped with software for data acquisition and analysis. Additional modules can be supplied	LC system consisting of modules, such as autosampler, degasser, isocratic/binary quaternary pump, column thermostat, and a UV detector connected with a computer equipped with software for data acquisition and analysis	LC system consisting of modules, such as autosampler, degasser, isocratic/binary quaternary pump, and a column thermostat connected to tandem mass spectrometer with an ion source controlled with a computer equipped with software for data acquisition and analysis. Furthermore, a nitrogen gas generator and vacuum pump
Cost of instrument, replacing parts, maintenance	low	high	very high
Instrument maintenance	simple and fast	time-consuming but not advanced	time-consuming and advanced
Amount of waste	small	large	medium
Method development	intermediate	intermediate	advanced
Instrument control	easy	easy	easy
Data interpretation	easy	medium	advanced
Cost of single analysis	low	medium	high
Analysis time	short (even <1min)	long (~25–60 min)	short (~7–15 min)
Sample preparation	can be omitted	multi-step	multi-step
Consumption of organic solvents	low (standards’ preparation, electrode surface modification)	very high (preparation of standards, samples, mobile phase)	high (preparation of standards, samples and mobile phase, cleaning of MS source)
Sensitivity	high	intermediate	high
Selectivity	poor (can be improved by the proper modification of the electrode surface)	high (provided by separation of analytes on column, proper choice of analytical wavelength, gradient of the mobile phase, sample pretreatment)	high (provided by selection of the precursor and product ions characteristic for the analyte)
Number of monitored analytes during a single analysis	~1–3	numerous	numerous
Amount of sample injected for a single analysis	≥100 µL	≥10 µL	≤10 µL
Miniaturization	YES	NO	NO

## Data Availability

Not applicable.

## References

[1] Takikawa O. (2005). Biochemical and medical aspects of the indoleamine 2,3-dioxygenase-initiated l-tryptophan metabolism. Biochem. Biophys. Res. Commun..

[2] Marszalek-Grabska M., Walczak K., Gawel K., Wicha-Komsta K., Wnorowska S., Wnorowski A., Turski W.A. (2021). Kynurenine emerges from the shadows–Current knowledge on its fate and function. Pharmacol. Ther..

[3] Badawy A., Namboodiri A.M., Moffett J.R. (2016). The end of the road for the tryptophan depletion concept in pregnancy and infection. Clin. Sci..

[4] Mellor A.L., Lemos H., Huang L. (2017). Indoleamine 2,3-Dioxygenase and Tolerance: Where Are We Now?. Front. Immunol..

[5] Badawy A.A.B. (2017). Kynurenine pathway of tryptophan metabolism: Regulatory and functional aspects. Int. J. Tryptophan Res..

[6] Cervenka I., Agudelo L.Z., Ruas J.L. (2017). Kynurenines: Tryptophan’s metabolites in exercise, inflammation, and mental health. Science.

[7] Sinclair L.V., Neyens D., Ramsay G., Taylor M.P., Cantrell D.A. (2018). Single cell analysis of kynurenine and System L amino acid transport in T cells. Nat. Commun..

[8] Novikov O., Wang Z., Stanford E.A., Parks A.J., Ramirez-Cardenas A., Landesman E., Laklouk I., Sarita-Reyes C., Gusenleitner D., Li A. (2016). An Aryl Hydrocarbon Receptor-Mediated Amplification Loop That Enforces Cell Migration in ER-/PR-/Her2- Human Breast Cancer Cells. Mol. Pharmacol..

[9] Bock K.W. (2020). Aryl hydrocarbon receptor (AHR) functions: Balancing opposing processes including inflammatory reactions. Biochem. Pharmacol..

[10] Turski M.P., Turska M., Paluszkiewicz P., Parada-Turska J., Oxenkrug G.F. (2013). Kynurenic Acid in the Digestive system—new Facts, new challenges. Int. J. Tryptophan Res..

[11] Morita T., Saito K., Takemura M. (2001). 3-Hydroxyanthranilic acid, an L-tryptophan metabolite, induces apoptosis in monocyte-derived cells stimulated by interferon-γ. Ann. Clin. Biochem..

[12] Zaher S.S., Germain C., Fu H., Larkin D.F.P., George A.J.T. (2011). 3-Hydroxykynurenine Suppresses CD4+ T-Cell Proliferation, Induces T-Regulatory-Cell Development, and Prolongs Corneal Allograft Survival. Investig. Ophthalmol. Vis. Sci..

[13] Guillemin G.J. (2012). Quinolinic acid, the inescapable neurotoxin. FEBS J..

[14] Malina H., Richter C., Frueh B., Hess O.M. (2002). Lens epithelial cell apoptosis and intracellular Ca2+ increasein the presence of xanthurenic acid. BMC Ophthalmol..

[15] Dhakar N.K., Caldera F., Bessone F., Cecone C., Pedrazzo A.R., Cavalli R., Dianzani C., Trotta F. (2019). Evaluation of solubility enhancement, antioxidant activity, and cytotoxicity studies of kynurenic acid loaded cyclodextrin nanosponge. Carbohydr. Polym..

[16] Chobot V., Hadacek F., Weckwerth W., Kubicova L. (2015). Iron chelation and redox chemistry of anthranilic acid and 3-hydroxyanthranilic acid: A comparison of two structurally related kynurenine pathway metabolites to obtain improved insights into their potential role in neurological disease development. J. Organomet. Chem..

[17] Sathyasaikumar K.V., Tararina M., Wu H.Q., Neale S.A., Weisz F., Salt T.E., Schwarcz R. (2017). Xanthurenic Acid Formation from 3-Hydroxykynurenine in the Mammalian Brain: Neurochemical Characterization and Physiological Effects. Neuroscience.

[18] Richard D.M., Dawes M.A., Mathias C.W., Acheson A., Hill-Kapturczak N., Dougherty D.M. (2009). L-Tryptophan: Basic Metabolic Functions, Behavioral Research and Therapeutic Indications. Int. J. Tryptophan Res..

[19] Savitz J. (2020). The kynurenine pathway: A finger in every pie. Mol. Psychiatry.

[20] Platten M., Nollen E.A.A., Röhrig U.F., Fallarino F., Opitz C.A. (2019). Tryptophan metabolism as a common therapeutic target in cancer, neurodegeneration and beyond. Nat. Rev. Drug Discov..

[21] Badawy A.A.-B. (2015). Tryptophan metabolism, disposition and utilization in pregnancy. Biosci. Rep..

[22] Bohár Z., Toldi J., Fülöp F., Vécsei L. (2015). Changing the face of kynurenines and neurotoxicity: Therapeutic considerations. Int. J. Mol. Sci..

[23] Opitz C.A., Wick W., Steinman L., Platten M. (2007). Tryptophan degradation in autoimmune diseases. Cell. Mol. Life Sci..

[24] Sheipouri D., Braidy N., Guillemin G.J. (2013). Kynurenine pathway in skin cells: Implications for UV-induced skin damage. Int. J. Tryptophan Res..

[25] Mehraj V., Routy J.-P. (2015). Tryptophan Catabolism in Chronic Viral Infections: Handling Uninvited Guests. Int. J. Tryptophan Res..

[26] Tan V.X., Guillemin G.J. (2019). Kynurenine Pathway Metabolites as Biomarkers for Amyotrophic Lateral Sclerosis. Front. Neurosci..

[27] Sadok I., Jędruchniewicz K., Rawicz-Pruszyński K., Staniszewska M. (2021). UHPLC-ESI-MS/MS Quantification of Relevant Substrates and Metabolites of the Kynurenine Pathway Present in Serum and Peritoneal Fluid from Gastric Cancer Patients—Method Development and Validation. Int. J. Mol. Sci..

[28] Erabi H., Okada G., Shibasaki C., Setoyama D., Kang D., Takamura M., Yoshino A., Fuchikami M., Kurata A., Kato T.A. (2020). Kynurenic acid is a potential overlapped biomarker between diagnosis and treatment response for depression from metabolome analysis. Sci. Rep..

[29] Wang J. (2002). Portable electrochemical systems. TrAC Trends Anal. Chem..

[30] Tajik S., Beitollahi H., Mohammadi S.Z., Azimzadeh M., Zhang K., Van Le Q., Yamauchi Y., Jang H.W., Shokouhimehr M. (2020). Recent developments in electrochemical sensors for detecting hydrazine with different modified electrodes. RSC Adv..

[31] Ramachandran R., Chen T.-W., Chen S.-M., Baskar T., Kannan R., Elumalai P., Raja P., Jeyapragasam T., Dinakaran K., Gnana Kumar G. (2019). peter A review of the advanced developments of electrochemical sensors for the detection of toxic and bioactive molecules. Inorg. Chem. Front..

[32] Özel R.E., Hayat A., Andreescu S. (2015). Recent Developments in Electrochemical Sensors for the Detection of Neurotransmitters for Applications in Biomedicine. Anal. Lett..

[33] Sadok I., Gamian A., Staniszewska M.M. (2017). Chromatographic analysis of tryptophan metabolites. J. Sep. Sci..

[34] Anesi A., Rubert J., Oluwagbemigun K., Orozco-Ruiz X., Nöthlings U., Breteler M.M.B., Mattivi F. (2019). Metabolic profiling of human plasma and urine, targeting tryptophan, tyrosine and branched chain amino acid pathways. Metabolites.

[35] Zhu W., Stevens A.P., Dettmer K., Gottfried E., Hoves S., Kreutz M., Holler E., Canelas A.B., Kema I., Oefner P.J. (2011). Quantitative profiling of tryptophan metabolites in serum, urine, and cell culture supernatants by liquid chromatography-tandem mass spectrometry. Anal. Bioanal. Chem..

[36] Sadok I., Rachwał K., Staniszewska M. (2019). Application of the optimized and validated LC–MS method for simultaneous quantification of tryptophan metabolites in culture medium from cancer cells. J. Pharm. Biomed. Anal..

[37] Tömösi F., Kecskeméti G., Cseh E.K., Szabó E., Rajda C., Kormány R., Szabó Z., Vécsei L., Janáky T. (2020). A validated UHPLC-MS method for tryptophan metabolites: Application in the diagnosis of multiple sclerosis. J. Pharm. Biomed. Anal..

[38] Badawy A.A.-B., Morgan C.J. (2010). Rapid Isocratic Liquid Chromatographic Separation and Quantification of Tryptophan and Six kynurenine Metabolites in Biological Samples with Ultraviolet and Fluorimetric Detection. Int. J. Tryptophan Res..

[39] Marrugo-Ramírez J., Rodríguez-Núñez M., Marco M.-P., Mir M., Samitier J. (2021). Kynurenic Acid Electrochemical Immunosensor: Blood-Based Diagnosis of Alzheimer’s Disease. Biosensors.

[40] Kato D., Kamata T., Sumimoto M. (2021). Electrochemical Detection of Tryptophan Metabolites via Kynurenine Pathway by Using Nanocarbon Films. Electroanalysis.

[41] Sadok I., Tyszczuk-Rotko K., Mroczka R., Kozak J., Staniszewska M. (2021). Improved Voltammetric Determination of Kynurenine at the Nafion Covered Glassy Carbon Electrode –Application in Samples Delivered from Human Cancer Cells. Int. J. Tryptophan Res..

[42] Sadok I., Tyszczuk-Rotko K., Mroczka R., Staniszewska M. (2020). Simultaneous voltammetric analysis of tryptophan and kynurenine in culture medium from human cancer cells. Talanta.

[43] Brooks E.L., Mutengwa V.S., Abdalla A., Yeoman M.S., Patel B.A. (2019). Determination of tryptophan metabolism from biological tissues and fluids using high performance liquid chromatography with simultaneous dual electrochemical detection. Analyst.

[44] Kubicova L., Hadacek F., Bachmann G., Weckwerth W., Chobot V. (2019). Coordination Complex Formation and Redox Properties of Kynurenic and Xanthurenic Acid Can A ect Brain Tissue Homeodynamics. Antioxidants.

[45] Karami P., Majidi M.R., Johari-Ahar M., Barar J., Omidi Y. (2017). Development of screen-printed tryptophan-kynurenine immunosensor for in vitro assay of kynurenine-mediated immunosuppression effect of cancer cells on activated T-cells. Biosens. Bioelectron..

[46] Singh R., Kashyap S., Kumar S., Abraham S., Gupta T.K., Kayastha A.M., Malhotra B.D., Saxena P.S., Srivastava A., Singh R.K. (2017). Excellent storage stability and sensitive detection of neurotoxin quinolinic acid. Biosens. Bioelectron..

[47] Huang C.-Y., O’Hare D., Chao I.-J., Wei H.-W., Liang Y.-F., Liu B.-D., Lee M.-H., Lin H.-Y. (2015). Integrated potentiostat for electrochemical sensing of urinary 3-hydroxyanthranilic acid with molecularly imprinted poly (ethylene-co-vinylalcohol). Biosens. Bioelectron..

[48] Yamamura S., Hoshikawa M., Dai K., Saito H., Suzuki N., Niwa O., Okada M. (2013). ONO-2506 inhibits spike–wave discharges in a genetic animal model without affecting traditional convulsive tests via gliotransmission regulation. Br. J. Pharmacol..

[49] Liu L., Chen Y., Zhang Y., Wang F., Chen Z. (2011). Determination of tryptophan and kynurenine in human plasma by liquid chromatography-electrochemical detection with multi-wall carbon nanotube-modified glassy carbon electrode. Biomed. Chromatogr..

[50] Mackay G.M., Forrest C.M., Stoy N., Christofides J., Egerton M., Stone T.W., Darlington L.G. (2006). Tryptophan metabolism and oxidative stress in patients with chronic brain injury. Eur. J. Neurol..

[51] Vaarmann A., Kask A., Mäeorg U. (2002). Novel and sensitive high-performance liquid chromatographic method based on electrochemical coulometric array detection for simultaneous determination of catecholamines, kynurenine and indole derivatives of tryptophan. J. Chromatogr. B.

[52] Li J., Li G. (1998). Identification of 3-Hydroxykynurenine and Xanthurenic Acid and Quantitation of 3-Hydroxykynurenine Transaminase Activity Using HPLC with Electrochemical Detection. J. Liq. Chromatogr. Relat. Technol..

[53] Iwahashi H., Ishii T. (1997). Detection of the oxidative products of 3-hydroxykynurenine using high-performance liquid chromatography–electrochemical detection–ultraviolet absorption detection–electron spin resonance spectrometry and high-performance liquid chromatography–electrochemi. J. Chromatogr. A.

[54] Malone M.A., Zuo H., Lunte S.M., Smyth M.R. (1995). Determination of tryptophan and kynurenine in brain microdialysis samples by capillary electrophoresis with electrochemical detection. J. Chromatogr. A.

[55] Pearson S.J., Reynolds G.P. (1992). Increased brain concentrations of a neurotoxin, 3-hydroxykynurenine, in Huntington’s disease. Neurosci. Lett..

[56] Beal M.F., Matson W.R., Storey E., Milbury P., Ryan E.A., Ogawa T., Bird E.D. (1992). Kynurenic acid concentrations are reduced in Huntington’s disease cerebral cortex. J. Neurol. Sci..

[57] Pearson S.J., Reynolds G.P. (1991). Determination of 3-hydroxykynurenine in human brain and plasma by high-performance liquid chromatography with electrochemical detection. Increased concentrations in hepatic encephalopathy. J. Chromatogr..

[58] Swartz K.J., Matson W.R., MacGarvey U., Ryan E.A., Beal M.F. (1990). Measurement of kynurenic acid in mammalian brain extracts and cerebrospinal fluid by high-performance liquid chromatography with fluorometric and coulometric electrode array detection. Anal. Biochem..

[59] Heyes M.P., Quearry B.J. (1988). Quantification of 3-hydroxykynurenine in brain by high-performance liquid chromatography and electrochemical detection. J. Chromatogr. B Anal. Technol. Biomed. Life Sci..

[60] Fukunaga M., Yamamoto Y., Kawasoe M., Arioka Y., Murakami Y., Hoshi M., Saito K. (2012). Studies on tissue and cellular distribution of indoleamine 2,3-dioxygenase 2: The absence of IDO1 upregulates IDO2 expression in the epididymis. J. Histochem. Cytochem..

[61] Kanai M., Nakamura T., Funakoshi H. (2009). Identification and characterization of novel variants of the tryptophan 2,3-dioxygenase gene: Differential regulation in the mouse nervous system during development. Neurosci. Res..

[62] Takikawa O., Yoshid R., Kido R., Hayaishi O. (1986). Tryptophan Degradation in Mice Initiated by Indoleamine 2,3-Dioxygenase. J. Biol. Chem..

[63] Fukui S., Schwarcz R., Rapoport S.I., Takada Y., Smith Q.R. (1991). Blood–Brain Barrier Transport of Kynurenines: Implications for Brain Synthesis and Metabolism. J. Neurochem..

[64] Goeden N., Notarangelo F.M., Pocivavsek A., Beggiato S., Bonnin A., Schwarcz R. (2017). Prenatal Dynamics of Kynurenine Pathway Metabolism in Mice: Focus on Kynurenic Acid. Dev. Neurosci..

[65] Pawlak D., Tankiewicz A., Matys T., Buczko W. (2003). Peripheral distribution of kynurenine metabolites and activity of kynurenine pathway enzymes in renal failure. J. Physiol. Pharmacol..

[66] Hiratsuka C., Sano M., Fukuwatari T., Shibata K. (2014). Time-dependent effects of L-tryptophan administration on urinary excretion of L-tryptophan metabolites. J. Nutr. Sci. Vitaminol. (Tokyo).

[67] Price J.M., Brown R.R., Peters H.A. (1959). Tryptophan metabolism in porphyria, schizophrenia, and a variety of neurologic and psychiatric diseases. Neurology.

[68] Galla Z., Rajda C., Rácz G., Grecsó N., Baráth Á., Vécsei L., Bereczki C., Monostori P. (2021). Simultaneous determination of 30 neurologically and metabolically important molecules: A sensitive and selective way to measure tyrosine and tryptophan pathway metabolites and other biomarkers in human serum and cerebrospinal fluid. J. Chromatogr. A.

[69] Kędzierski W., Sadok I., Kowalik S., Janczarek I., Staniszewska M. (2021). Does the type of exercise affect tryptophan catabolism in horses?. Anim. Int. J. Anim. Biosci..

[70] Bizzarri M., Catizone A., Pompei M., Chiappini L., Curini L., Lagana A. (1990). Determination of urinary tryptophan and its metabolites along the nicotinic acid pathway by high performance liquid chromatography with ultraviolet detection. Biomed. Chromatogr..

[71] Buczko P., Stokowska W., Górska W., Kucharewicz I., Pawlak D., Buczko W. (2006). Tryptophan Metabolites via Kynurenine Pathway in Saliva of Diabetic Patients. Dent. Med. Probl..

[72] Baran H., Jellinger K., Deecke L. (1999). Kynurenine metabolism in Alzheimer’s disease. J. Neural Transm..

[73] Wang L.-S., Zhang M.-D., Tao X., Zhou Y.-F., Liu X.-M., Pan R.-L., Liao Y.-H., Chang Q. (2019). LC-MS/MS-based quantification of tryptophan metabolites and neurotransmitters in the serum and brain of mice. J. Chromatogr. B Anal. Technol. Biomed. Life Sci..

[74] Yamada K., Miyazaki T., Shibata T., Hara N., Tsuchiya M. (2008). Simultaneous measurement of tryptophan and related compounds by liquid chromatography/electrospray ionization tandem mass spectrometry. J. Chromatogr. B Anal. Technol. Biomed. Life Sci..

[75] Tkaczuk-Włach J., Kędzierski W., Jonik I., Sadok I., Filip A., Kankofer M., Polkowski W., Ziółkowski P., Gamian A., Staniszewska M. (2021). Immunomodulatory Factors in Primary Endometrial Cell Cultures Isolated from Cancer and Noncancerous Human Tissue –Focus on RAGE and IDO1. Cells.

[76] Christen S., Thomas S.R., Garner B., Stocker R. (1994). Inhibition by interferon-gamma of human mononuclear cell-mediated low density lipoprotein oxidation. Participation of tryptophan metabolism along the kynurenine pathway. J. Clin. Investig..

[77] Bonda D.J., Mailankot M., Stone J.G., Garrett M.R., Staniszewska M., Castellani R.J., Siedlak S.L., Zhu X., Lee H., Perry G. (2010). Indoleamine 2,3-dioxygenase and 3-hydroxykynurenine modifications are found in the neuropathology of Alzheimer’s disease. Redox Rep..

[78] Mitsuhashi S., Fukushima T., Tomiya M., Santa T., Imai K., Toyo’oka T. (2007). Determination of kynurenine levels in rat plasma by high-performance liquid chromatography with pre-column fluorescence derivatization. Anal. Chim. Acta.

[79] Zhao J., Gao P., Zhu D. (2010). Optimization of Zn2+-containing mobile phase for simultaneous determination of kynurenine, kynurenic acid and tryptophan in human plasma by high performance liquid chromatography. J. Chromatogr. B.

[80] Zhou W., Yang S., Wang P.G. (2017). Matrix effects and application of matrix effect factor. Bioanalysis.

[81] Naritsin D.B., Bonl R.L., Markey S.P. (1995). Pentafluorobenzylation Method for Quantification of Acidic Tryptophan Metabolites Using Electron Capture Negative Ion Mass Spectrometry. Anal. Chem..

[82] Smythe G.A., Braga O., Brew B.J., Grant R.S., Guillemin G.J., Kerr S.J., Walker D.W. (2002). Concurrent Quantification of Quinolinic, Picolinic, and Nicotinic Acids Using Electron-Capture Negative-Ion Gas Chromatography–Mass Spectrometry. Anal. Biochem..

[83] Chawdhury A.S.M.M.R., Shamsi S.A., Miller A., Liu A. (2021). Capillary electrochromatography-mass spectrometry of kynurenine pathway metabolites. J. Chromatogr. A.

[84] Cannazza G., Baraldi M., Braghiroli D., Tail A., Parenti C. (2003). High-performance liquid chromatographic method for the quantification of anthranilic and 3-hydroxyanthranilic acid in rat brain dialysate. J. Pharm. Biomed. Anal..

[85] Singh R., Kashayap S., Singh V., Kayastha A.M., Mishra H., Saxena P.S., Srivastava A., Singh R.K. (2017). QPRTase modified N-doped carbon quantum dots: A fluorescent bioprobe for selective detection of neurotoxin quinolinic acid in human serum. Biosens. Bioelectron..

[86] Mawatari K., Iinuma F., Watanabe M. (1990). Fluorometric determination of urinary kynurenic acid by flow injection analysis equipped with a “bypass line”. Anal. Biochem..

[87] Chen C.-F., Liu T.-Z., Lan W.-H., Wu L.-A., Tsai C.-H., Chiou J.-F., Tsai L.-Y. (2013). Novel Spectrophotometric Method for the Quantitation of Urinary Xanthurenic Acid and Its Application in Identifying Individuals with Hyperhomocysteinemia Associated with Vitamin B6 Deficiency. Biomed Res. Int..

[88] Park A., Yang Y., Lee Y., Kim M.S., Park Y.-J., Jung H., Kim T.-D., Lee H.G., Choi I., Yoon S.R. (2019). Indoleamine-2,3-Dioxygenase in Thyroid Cancer Cells Suppresses Natural Killer Cell Function by Inhibiting NKG2D and NKp46 Expression via STAT Signaling Pathways. J. Clin. Med..

[89] Li G., Miao P. (2013). Theoretical Background of Electrochemical Analysis. Electrochemical Analysis of Proteins and Cells. SpringerBriefs in Molecular Science.

[90] Muzyka K., Sun J., Fereja T.H., Lan Y., Zhang W., Xu G. (2019). Boron-doped diamond: Current progress and challenges in view of electroanalytical applications. Anal. Methods.

[91] Maiyalagan T., Pasupathi S. (2010). Components for PEM Fuel cells: An Overview. Mater. Sci. Forum.

[92] Hashemi B., Zohrabi P., Shamsipur M. (2018). Recent developments and applications of different sorbents for SPE and SPME from biological samples. Talanta.

[93] Suprun E.V., Karpova E.V., Radko S.P., Karyakin A.A. (2020). Advanced electrochemical detection of amino acids and proteins through flow injection analysis and catalytic oxidation on Prussian Blue. Electrochim. Acta.

[94] Hamzah H.H., Zain Z.M., Musa N.L.W., Lin Y.-C., Trimbee E. (2013). Spectrophotometric Determination of Uric Acid in Urine Based-Enzymatic Method Uricase with 4-Aminodiphenylamine Diazonium Sulfate (Variamine Blue RT Salt). J. Anal. Bioanal. Tech..

[95] Kong D., Zhuang Q., Han Y., Xu L., Wang Z., Jiang L., Su J., Lu C.-H., Chi Y. (2018). Simultaneous voltammetry detection of dopamine and uric acid in human serum and urine with a poly(procaterol hydrochloride) modified glassy carbon electrode. Talanta.

[96] Pelletier O. (1968). Determination of vitamin C in serum, urine, and other biological materials. J. Lab. Clin. Med..

[97] Harris A.R., Carter P., Cowan R., Wallace G.G. (2021). Impact of Protein Fouling on the Charge Injection Capacity, Impedance, and Effective Electrode Area of Platinum Electrodes for Bionic Devices. ChemElectroChem.

[98] Downard A.J., Roddick A.D. (1995). Protein Adsorption at Gl.assy Carbon Electrodes: The Effect of Covalently Bound Surface Groups. Electroanalysis.

[99] Beykal B., Herzberg M., Oren Y., Mauter M.S. (2015). Influence of surface charge on the rate, extent, and structure of adsorbed Bovine Serum Albumin to gold electrodes. J. Colloid Interface Sci..

[100] Žurga N., Majer D., Finšgar M. (2021). Pb(II) Determination in a Single Drop Using a Modified Screen-Printed Electrode. Chemosensors.

[101] Ferrari A.G.-M., Rowley-Neale S.J., Banks C.E. (2021). Screen-printed electrodes: Transitioning the laboratory in-to-the field. Talanta Open.

[102] Lipkowski J. (2011). Challenges and opportunities of modern electrochemistry—a personal reflection. J. Solid State Electrochem. Vol..

[103] Teymourian H., Parrilla M., Sempionatto J.R., Montiel N.F., Barfidokht A., Van Echelpoel R., De Wael K., Wang J. (2020). Wearable Electrochemical Sensors for the Monitoring and Screening of Drugs. ACS Sens..

